# CT imaging history for patients presenting to the ED with renal colic--evidence from a multi-hospital database

**DOI:** 10.1186/s12873-019-0232-7

**Published:** 2019-03-01

**Authors:** Emily Schmid, Kimberly Leeson, K. Tom Xu, Peter Richman, Crystal Nwosu, Lynn Carrasco

**Affiliations:** 1 Department of Emergency Medicine, CHRISTUS Health/Texas A&M School of Medicine, Corpus Christi, TX USA; 2 Texas Tech University School of Medicine, Bullock, Lubbock, TX USA; 3 CHRISTUS HEALTH/Texas A&M Residency in Emergency Medicine, 600 Elizabeth Street, Corpus Christi, TX 78404 USA

**Keywords:** Renal colic, Repeat imaging, CT scan

## Abstract

**Background:**

Patients with renal colic have a 7% chance of annual recurrence. Previous studies evaluating cumulative Abbreviations: computed tomography (CT) exposure for renal colic patients were typically from single centers.

**Methods:**

This was an observational cohort study. Inner-city ED patients with a final diagnosis of renal colic were prospectively identified (1/10/16–10/16/16). Authors conducted structured electronic record reviews from a 6-hospital system encompassing over 192,000 annual ED visits. Categorical data analyzed by chi-square; continuous data by t-tests. Primary outcome measure was the proportion of study group patients with prior history CT abdomen/pelvis CT.

**Results:**

Two hundred thirteen patients in the study group; 59% male, age 38+/− 10 years, 67% Hispanic, 62% prior stone history, flank pain (78%), dysuria (22%), UA (+) blood (75%). 60% (95% CI = 53–66%) of patients received an EDCV CT; hydronephrosis seen in 55% (95% CI = 46–63%), stone in 90%(95% CI = 83–94%). No significant differences observed in the proportion of EDCV patients who received CT with respect to: female vs. male (62% vs. 56%; *p* = 0.4), mean age (37+/− 9 years vs. 39+/− 11 years; *p* = 0.2), and Hispanic vs. non-Hispanic white (63% vs.63%; *p* = 0.96). Patients with a prior stone history were more likely than those with no history to receive an EDCV CT (88% vs. 16%; *p* < 0.001). 118 (55%; 95% CI = 49–62%) of patients had at least one prior CT, 46 (22%; 95% CI = 16–28%) had ≥3 prior CTs; 29 (14%; 95% CI = 10–19%), ≥ 10 prior CTs. Patients who did not receive an EDCV CT had a significantly higher mean prior number of CTs than those who had EDCV CT (5.1+/− 7.7 vs 2.2+/− 4.9; p < 0.001). Patients with prior stone were more likely to receive only U/S during EDCV (33% vs. 15%; *p* = 0.003).

**Conclusions:**

Within our EDCV cohort of renal colic patients, 55% had at least one prior CT. The mean number of prior CTs was lower for patients receiving CT on EDCV, and Ultrasound (US) alone was used more often in patients with prior stone history vs. those with no prior history.

## Background

Numerous investigators have raised concerns regarding the potential relationship between ionizing radiation dose from medical imaging and risk of human malignancy [1–5]. Depending on the area imaged, a single CT scan is estimated to increase the lifetime risk of cancer by as much as 1 in 2000 [3]. Despite such risk, the use of CT has grown significantly over the past 20 years to evaluate for pulmonary embolism and a variety of intra-abdominal conditions. The average growth rate of CT utilization for chest pain complaints alone was 28.1% per year between 2001 and 2007 [6].

Several publications in the mid-1990s touted the use of CT to accurately identify the presence of nephrolithiasis and alternative conditions in ED patients with suspected renal colic. Within just a few years, unenhanced CT of the abdomen and pelvis supplanted intravenous pyelography (IVP) as the diagnostic study of choice for evaluating patients with suspected renal colic. [7] Similar to the observed trends for chest imaging, CT utilization for the evaluation of patients with flank pain grew exponentially [7, 8]. Westphalen et al. found that from 1996 to 2007 there was a 10-fold increase in CT scan use for patients with suspected kidney stone, but the authors found no associated change in the proportion of diagnosis of kidney stone, diagnosis of significant alternate diagnoses, or admission to the hospital [7]. A similar study conducted from 2000 to 2008 revealed a significant increase in computerized tomography use, a significant decrease in x-ray use, historically the preferred imaging study for this condition, while ultrasound use remained stable. They also found that despite the increased use of CT scan, the proportion of patients diagnosed with kidney stone did not increase [8].

The growing use of CT scan as the diagnostic modality of choice in renal colic patients is particularly concerning because of the recurrent nature of the condition. The average patient with renal colic has a 7% chance of recurrence per year and a 50% chance of a recurrence within 10 years [9]. A single center, retrospective chart review including 356 patient encounters over a 10-month period by Broder et al. found that 79% of patients presenting to emergency departments with recurrent symptoms suggesting renal colic underwent at least 2 CT scans [10]. Katz et al. estimated that a typical single detector unenhanced CT and single multi-detector unenhanced CT of the abdomen and pelvis exposes a patient to 6.5 mSv and 8.5 mSv respectively [5]. Unfortunately, within their single-center cohort of 4562 patients undergoing repeat CT scan for suspected renal colic, they found patients with three or more unenhanced CT scans of the abdomen and pelvis were exposed to ionizing radiation doses ranging from 20 to 154 mSv. Such exposure falls within a range that Brenner et al. found suggestive of significantly increased cancer risks (10–50 mSv for an acute exposure and 50–100 mSv for a protracted exposure) [4].

Prior studies related to CT scan use in renal colic have been mostly small, single center studies or have relied on national, administrative databases [5, 7–15]. We are unaware of prospective emergency department-based studies, to date, that evaluated repeated patient exposure for renal colic imaging within a multi-center database. The objective of our study was to evaluate whether our patients had been exposed to multiple CTs for the evaluation of renal colic within a regional hospital system and to identify patient characteristics that were associated with CT use during the current ED visit.

## Methods

### Study design

This was a multi-center prospective observational cohort study of inner-city patients designed to compare characteristics and imaging history from a six-hospital database for a cohort of patients diagnosed with renal colic during their current ED visit (EDCV). Subjects were verbally consented for participation.

### Setting

Patients were enrolled that presented to CHRISTUS Spohn Memorial hospital emergency department (Corpus Christi, Texas). The facility is a teaching affiliate of the Texas A&M Health Science Center, a level-two trauma center, and serves an inner-city population. The annual Emergency Department census is 45,000 patients. The study was approved by the CHRISTUS Spohn Institutional Review Board prior to initiation of patient enrollment.

### Population

Adult patients greater than 18 years of age that received a diagnosis of renal colic at their current ED visit were eligible for enrollment. The CHRISTUS Spohn Institutional Review Board provided a waiver of consent as all data was available in the medical record. Patients less than 18 years of age and pregnant patients were excluded from the study.

### Study protocol

CHRISTUS Spohn Memorial emergency physicians with the aid of trained research associates prospectively identified the patient cohort over a 10-month period from January 10, 2016 to October 16, 2016 at time of care. Demographic and historical features were recorded on a data collection form. To determine prior imaging exposure and CT results for patients, the study authors conducted structured chart reviews of a shared electronic medical record (EMR) for a 6-hospital system, including 192,073 annual ED visits (70.6% of all ED visits within a twelve-county region). The study also evaluated the method by which the patient was diagnosed with renal colic at the current ED visit, including laboratory and imaging results.

### Statistical methods

Categorical data was analyzed by chi square and 95% confidence intervals were calculated. Continuous data was analyzed by t-tests. All tests were two-tailed; alpha set at 0.05. The primary outcome parameter was to observe the proportion of patients with renal colic who had a history of multiple CTs for renal colic within our system. Secondary outcomes included identifying patients’ characteristics that were associated recurrent CT use, CT use for the current visit, and ultrasound use for the current visit.

## Results

Patient characteristics are shown in Table 1. Of the 213 patients included in the final study group, 59% were male and 67% were Hispanic. The mean age was 38 +/− 11 years. Sixty-two percent of patients reported a prior history of kidney stones. The most common presenting symptom was flank pain and hematuria was detected by urinalysis in 76% of patients (Table 2).

**Table 1:** Presenting signs, symptoms, and laboratory findings

Flank pain	78%
Abdominal pain	50%
Nausea and/or vomiting	62%
Dysuria	22%
Blood on urinalysis	76%

**Table 2:** Patient prior CT scans of the abdomen/pelvis

Number of prior CT scans	Frequency	Percent
0	95	45
1	46	22
2	17	8
3	11	5
4	6	3
5	2	1
6	1	0.5
7	6	3
12	1	0.5
13	12	6
14	3	1
20	1	0.5
23	2	1
24	5	2
25	4	2
26	1	0.5

Table 3 reveals that 60% (95% CI = 53–66%) of patients received a CT scan at the ED current visit and 30% of patients underwent US. Only 4% of patients underwent both a CT and an US and 14% of patients had no imaging performed at their current visit. Hydronephrosis was present on 55% (95% CI = 46–63%) of CTs and 28% (95% CI = 19–40%) of ultrasounds performed during the current visit. A renal stone was identified on 90% (95% CI = 83–94%) of CTs and 38% (95% CI = 45–74%) of ultrasounds performed in those cases.

**Table 3:** Current visit imaging

CT	60%
US	30%
US only	27%
CT only	56%
No imaging	14%
CT and US	4%

No significant differences were observed in the proportion of patients for the current ED visit who received CT with respect to: female vs. male (62% vs. 56%; *p* = 0.4), mean age (37+/− 9 years vs. 39+/− 11 years; *p* = 0.2), and Hispanic vs. non-Hispanic white (63% vs.63%; *p* = 0.96). Patients with a prior stone history were more likely than those with no history to receive an EDCV CT (88% vs. 16%; *p* < 0.001). Patients with a urinalysis positive for blood were no more likely to undergo CT than patients with no blood on urinalysis (61% vs. 60%; p = 0.4).

Table 4 shows patients’ prior CT histories. Out of the 213 patients, 118 (55%; 95% CI = 49–62%) had at least one prior CT, 46 (22%; 95% CI = 16–28%) had ≥3 prior CTs, and 29 patients (14%; 95% CI = 10–19%) had ≥10 prior CTs. Patients who did not receive a CT at the current visit had a significantly higher mean prior number of CTs than those who had current visit CT (5.1+/− 7.7 vs 2.2+/− 4.9; *p* < 0.001). Patients who received an US at their current visit also had a significantly higher mean number of prior CTs (4.9+/− 7.6 vs 2.8+/− 5.6; p < 0.001). Characteristics associated with positive prior CT history included: female gender (42% vs. 28%; *p* = 0.02), flank pain (40% vs. 13%; p < 0.001), and prior renal stone by history (48% vs. 10%; p < 0.001).

**Table 4:** Patient prior CT scans of the abdomen/pelvis

Number of prior CT scans	Frequency	Percent
0	95	45
1	46	22
2	17	8
3	11	5
4	6	3
5	2	1
6	1	0.5
7	6	3
12	1	0.5
13	12	6
14	3	1
20	1	0.5
23	2	1
24	5	2
25	4	2
26	1	0.5

Patients with a reported prior stone were more likely to receive a renal ultrasound as the only imaging study during the EDCV (33% vs. 15%; *p* = 0.003). Patients with a history of one or more CT scans were also more likely to undergo only an ultrasound compared to those with no prior CT (35% vs. 22%; *p* = 0.046). For patients who received only an US at their current visit, there were no significant differences observed with respect to female vs. male (26% vs. 26%; *p* = 1.0), mean age (37+/− 9 vs. 38+/− 11), or Hispanic vs. non-Hispanic white (23% vs. 25%;*p* = 0.4).

## Discussion

Unenhanced CT is a rapid and highly accurate imaging modality with reported specificity and sensitivity between 91 and 100% for diagnosing nephrolithiasis in patients presenting with flank pain [12, 13]. In theory, physicians and their patients benefit from CT use as it may also identify alternative significant diagnoses. However, somewhat surprisingly, the exponential increase in the use of CT scan for evaluation of flank pain has not resulted in significant improvements such as finding alternate diagnoses, better diagnosis of kidney stone, changing rates of admissions to the hospital, and/or identifying those in need of immediate urologic intervention [7, 14].

Several recent studies propose that renal ultrasound should be used as the first or even only diagnostic study when evaluating renal colic. The advantages of ultrasound include a low cost and widespread availability [16]. Investigators have reported that utilizing ultrasound as the initial diagnostic study of choice for renal colic is associated with lower cumulative radiation exposure without significant differences in complications, serious adverse events, pain scores, return emergency department visits, or hospitalizations [15]. In addition, studies comparing CT and US have shown a specificity of 95–100% and sensitivity between 61 and 93% for diagnosing ureterolithisis with US. These results suggest that US may be the ideal screening test, while CT is be reserved for situations when US is unavailable or non-diagnostic [12, 13].

Another proposed benefit to using CT is the ability to accurately measure stone size and degree of urinary obstruction. CT can also provide information about the need for further urologic intervention such as stenting [9]. However, authors of a recent study observed that the vast majority of patients receiving a CT scan as part of their work-up for renal colic did not require admission or immediate intervention for their kidney stone. Thus, there is a large population of low risk patients with suspected renal stones that could safely be managed with either ultrasound alone, expectant management or delayed CT [14]. Unfortunately, despite evidence that ultrasound is a cost-effective and useful alternative without inherent radiation risks, CT utilization continues to be the prevalent modality for diagnosis of ureterolithiasis and renal colic in the ED [7, 8, 10, 14].

We believe that our study is novel for utilizing a multi-hospital database across a geographic region to determine the prior imaging exposure of our ED patients. While surely not inclusive of all prior studies that might have been obtained at free-standing and non-system hospitals, our hospital system captures the majority of ED visits in a 12-county region. Further, we suggest that our study adds to the current understanding of imaging patterns for ED patients as we examined patient characteristics and clinical history that was associated with CT use both at the current visits and past visits respectively.

Our study demonstrated a lower rate of CT scanning in this population than that which has been reported in other similar studies. (9, 12–15) We observed that during the current visit, 60% of all patients presenting with suspected renal colic underwent CT imaging as part of their diagnostic work-up. Within our cohort, patients reporting a history of kidney stones were much more likely to undergo a CT scan on the current visit than those with no reported kidney stone history (88% versus 16%; *p* < 0.001). Disturbingly, within our multi-hospital system EMR, we found that 55% of all enrolled patients had at least one prior CT, 22% had 3 or more prior CTs, and 14% had 10 or more CTs.

On the other hand, we report findings that suggest that our physicians may have been aware of the patients’ imaging history. Patients who did not receive a current ED visit CT had a significantly higher mean prior number of CTs than those who underwent a CT scan at the current visit (5.1+/− 7.7 vs 2.2+/− 4.9; p < 0.001). This notion is further supported by our observation that patients with a prior stone history were much more likely to receive only a renal US during the EDCV (33% vs. 15%; *p* = 0.003).

## Limitations and future questions

Our study has several limitations that warrant discussion. Our study population was predominantly Hispanic and from lower socioeconomic groups. Thus, our findings may not be generalizable to other settings. As we noted previously, our methodology likely underestimates the prior imaging exposure of our patients as we cannot account for CTs performed outside of our hospital system. Regardless of such a limitation, our records identified a large percentage of patients with a history of recurrent imaging, so the findings are, nonetheless concerning. Finally, although our data suggest some reason for hope that the practice of emergency physicians may be evolving toward primary use of ultrasound for work-up of renal colic, our practice setting may not be generalizable to others as we have a teaching environment with residents and clinical faculty providing care to patients. Future investigators should examine the evolution of imaging practice for ED renal colic evaluation in other types of institutions and for other patient populations.

## Conclusions

Within our ED cohort of renal colic patients, 55% had at least one prior CT. The mean number of prior CTs was lower for patients receiving CT on EDCV, and Ultrasound (US) alone was used more often in patients with prior stone history vs. those with no prior history.

## Abbreviations

CT: Computed tomography
EDCV: Emergency department visit
IVP: Intravenous pyelography
US: Ultrasound
